# Digital Technologies for Children With Hearing Impairments to Support Language Learning: Scoping Review

**DOI:** 10.2196/85066

**Published:** 2026-06-16

**Authors:** Amarria Dila Sari, Abdullah Al Mahmud, Johanna Renny Octavia, Shivani Tyagi

**Affiliations:** 1 Centre for Design Innovation, Department of Architectural and Industrial Design, Swinburne University of Technology, John St, Hawthorn, Melbourne, 3122, Australia, 61 392143830; 2 Department of Industrial Engineering, Universitas Islam Indonesia, Yogyakarta, Indonesia; 3 Centre for Ergonomics, Parahyangan Catholic University, Bandung, Indonesia

**Keywords:** assistive technologies, digital technologies, augmentative and alternative communication, information communication technology, scoping review, children, digital health intervention, youth, hearing impairment, hearing loss, deaf, hard of hearing

## Abstract

**Background:**

Children with hearing impairments (HIs) have traditionally faced difficulties with language acquisition due to various factors, such as difficulties in accessing early intervention and therapy, among others. There is an opportunity for digital technology to address this problem; however, how different technologies can facilitate language acquisition, as well as the level of evidence, is not well understood.

**Objective:**

This study aimed to explore the types, roles, and outcomes of different digital technologies in supporting language development in children with HIs.

**Methods:**

Following Arksey and O’Malley’s framework for conducting a scoping review and the PRISMA-ScR (Preferred Reporting Items for Systematic Reviews and Meta-Analyses Extension for Scoping Reviews), we systematically searched 6 online databases from 2014 to September 2024 for relevant studies on the role of digital technologies in supporting language learning among children with HIs (aged <18 years).

**Results:**

A total of 45 studies met the inclusion criteria. Mobile apps were the most frequently reported technologies, followed by digital books, extended reality, computer-based programs, and tangible or robotic tools. The most commonly used signed-language apps focus on vocabulary development and visual communication. Spoken language tools were less common but included augmentative and alternative communication (AAC) systems and auditory training apps. The efficacy outcomes of 12 studies included in this review showed that AAC-based apps had stronger effects on expressive language, pragmatic communication skills, and vocabulary development. These outcomes occurred when AAC-based apps were used in structured contexts with a therapist or parent. In contrast, signed language apps often reported narrower, word-level outcomes. Notably, relatively few interventions targeted early spoken language development, despite early childhood being a critical period for language acquisition. In addition, limited attention was given to visual design considerations related to user comfort, cultural relevance, and contextual adaptation.

**Conclusions:**

The evidence base reveals 3 structural patterns: the dominance of accessibility-driven mobile technologies; an imbalance between signed and spoken-language interventions, characterized by a greater number of signed-language tools but stronger and more consistent evidence for spoken-language outcomes, particularly in AAC-based interventions; and limited integration of digital technologies across home, school, and clinical contexts. Future research should prioritize the co-design and evaluation of culturally responsive, integrated, developmentally appropriate digital systems that support early spoken-language development and sustained, family-mediated language learning across contexts.

## Introduction

### Background

Globally, more than 5% of people worldwide, about 430 million, including 34 million children, are living with disabling hearing loss, and this number is expected to rise to more than 700 million by 2050 [1]. Children with hearing impairments (HIs) encounter considerable challenges in acquiring spoken-language skills. HI refers to a reduced ability to hear sounds at normal levels, with varying degrees of severity. In children, hearing loss is typically diagnosed when the pure tone average exceeds 15 dB across key frequencies [2]. The scope of HI ranges from mild to profound. Children with profound hearing loss, who are often referred to as deaf, are unable to hear conversational speech without hearing technology, such as hearing aids or cochlear implants [2,3]. Those with mild to moderately severe hearing loss, commonly referred to as being hard of hearing, can often benefit from hearing aids [4]. In the context of this review, the term “children with HI” is used to refer to children who are deaf, hard of hearing, or have varying levels of hearing loss. Where relevant, cultural terms such as “deaf” have been used as per their application in the literature.

Communication preferences of children with HIs vary based on their access to hearing devices and language training. Some children rely on signed languages, while others use auditory input to develop spoken language through devices such as cochlear implants or hearing aids [5]. However, even with assistive devices, many children experience persistent challenges in developing spoken language due to limited access to early intervention, variability in device effectiveness, and insufficient support at home or school, which can lead to academic, behavioral, and social difficulties [6]. These challenges are compounded by barriers such as limited access to therapy, high costs, and a lack of parental knowledge regarding effective language-learning methods [7-9]. Many parents lack access to appropriate materials and training, leaving them unprepared to support their child’s language development [10]. Language learning can be defined as the process by which individuals develop the capacity to understand, create, and use language for communication purposes. This involves the acquisition of listening, speaking, reading, and writing skills, as well as the understanding of grammar, vocabulary, and social use of language [11]. Language learning progresses from basic abilities, which include auditory or visual awareness, to the acquisition of language skills, followed by the use of language for pragmatic purposes [12].

Assistive and digital tools offer a promising solution to address these challenges. According to ISO 9999:2016, assistive products are defined as tools, whether custom-made or commercially available, designed to support people with disabilities. These technologies, including mobile apps and tangible devices, are innovative ways to enhance spoken-language learning for children with HIs. These technologies provide accessible, cost-effective resources that can support both parents and therapists in speech therapy [8,13].

Recent studies have included augmentative and alternative communication (AAC) devices [14], virtual reality (VR) apps such as BEARS (Both Ears) developed by Vickers et al [15], and digital books designed for children with HIs [16]. Despite the potential of these tools, there remains a gap in the development of cost-effective, interactive technologies that children can use independently, with parental support [8,9,13,17]. In particular, mobile technology has tremendous potential for providing home-based auditory training and speech therapy [18].

Recent reviews have explored various digital tools for supporting children with HIs. Herrera et al [13] highlighted that using hearing technology in mobile learning for children was affordable and accessible, although efficacy analyses and attention to visual design were lacking. Frisby et al [19] reviewed 146 studies of mobile hearing health care services across all age groups, with most studies originating in high- and upper-middle-income countries. While the review highlighted a focus on screening, diagnosis, and treatment, it placed limited emphasis on language learning for children with HIs. Hajesmaeel-Gohari et al [20] investigated digital games and found that they could increase motivation; however, their therapeutic impact remains unclear. Similarly, Nanjundaswamy et al [21] reviewed computer-based auditory training programs and acknowledged a range of tools for different age groups, including children, but highlighted a critical lack of evidence proving their efficacy. These findings point to an important gap: despite the increasing presence of digital tools, there is a limited understanding of how they influence the language-learning outcomes of children with HIs, particularly those who rely on spoken language. This scoping review addresses this gap by mapping the landscape of assistive and digital technologies for children with HIs, examining their features, contexts of use, and reported outcomes. In particular, the extent to which these tools support spoken-language development, for which evidence is scarce but is urgently needed, is explored.

### Aim

This scoping review seeks to establish the landscape of assistive and digital technologies used to facilitate language learning among children with HIs. The review covers the following areas: (1) types of digital technologies applied, (2) the fundamental design elements and purposes of these technologies in facilitating language learning among children with HIs, and (3) the scope and nature of the reported efficacy outcomes. Although the review focuses on language learning as the core area of analysis, some studies that integrate language technologies with other domains of learning, such as literacy, mathematics, science, and general classroom interaction, are also included if language development remains a core component. The review covers both spoken language and sign language technologies to accommodate the varied communication needs of children with HIs. In this review, we have defined technology-based language learning interventions as digital tools intentionally designed to aid individuals in learning or improving language skills such as vocabulary acquisition, speech production, phonological awareness, listening comprehension, or signed language proficiency. Technologies whose primary purpose was unrelated to language learning, such as general communication aids or educational games, were included in this review only when the intervention explicitly targeted measurable language-learning outcomes.

## Methods

### Overview

This scoping review was conducted following the framework proposed by Arksey and O’Malley [22], which posits that scoping studies are useful for mapping key concepts, types of evidence, and gaps in the research within a given field, particularly when the area is complex or has not yet been comprehensively reviewed. In light of the variety of digital technologies as well as the research designs that have been implemented so far, this study does not intend to critically discuss the efficacy of specific interventions. Instead, it maps and summarizes the types of efficacy outcomes across studies. This design enabled the identification of patterns across the literature, highlighted areas with limited evidence, and informed recommendations for future research.

We used the PRISMA-ScR (Preferred Reporting Items for Systematic Reviews and Meta-Analyses Extension for Scoping Reviews; refer to Multimedia Appendix 1 for the complete Checklist 1) to guide the reporting in this scoping review [23] and developed a population, concept, and context framework [24] that is provided in Table 1 below. As this was a scoping review, no formal critical appraisal or statistical synthesis was performed.

**Table 1.** Population, concept, and context (PCC) framework.

PCC^a^ aspect	Description
Population	Children aged <18 years with HIs^b^, including children who are deaf, Deaf, or hard of hearing, as defined by the original studies.
Concept	Assistive and digital technology used to support language learning.
Context	Settings in which digital technology was used in controlled interventions to support the spoken-language learning of children with HIs. In addition to the contexts of intervention (home, school, or clinic), the geographical contexts (high-income countries or low- and middle-income countries) were also explored in descriptive terms under the component of “Context” in the PCC framework. However, this is only to look at the differences in access, scale, or learning environments and not to pose another research question in analytical terms.

a PCC: population, concept, and context.

b HI: hearing impairment.

### Step 1: Identifying the Research Questions (RQs)

This review is based on the following research questions that seek to explore how digital technologies are used in facilitating language learning and academic engagement for children with HIs:

RQ1. What types of digital technologies support language learning for children with HIs?

RQ2. What is the role of digital technology in supporting language learning and language-mediated academic participation among children with HIs?

RQ3. What are the reported efficacy outcomes of these digital technologies in supporting language development for children with HIs?

### Step 2: Search Strategy

The authors conducted a comprehensive search strategy to include all relevant literature published between January 2014 and September 2024 across the following databases: Ovid MEDLINE, PsycINFO, Web of Science, Scopus, CINAHL, and Computers and Applied Sciences Complete. An experienced librarian assisted in refining the subject headings and keywords to ensure broad coverage of technology-based interventions and HIs among children. The final search strategies are provided in Multimedia Appendix 1.

### Step 3: Study Selection

Studies needed to focus on the identification, implementation, and evaluation of assistive technology and digital technology for children with HIs to be included in this review. Refer to Textbox 1 for details regarding the inclusion and exclusion criteria. Eligible studies were identified using a 3-step process. In step 1, one reviewer (ADS) collected the search results and then removed duplicates. In step 2, two reviewers (ADS and AAM) reviewed the titles and abstracts of the identified studies that appeared to meet the inclusion criteria. In step 3, the same reviewers evaluated the full texts of the remaining publications that were identified in our searches to include potentially relevant publications. Covidence software (Veritas Health Innovation Ltd) was used to facilitate the screening, selection, and data management processes during the scoping review [25].

#### Eligibility Criteria

Studies needed to focus on the identification, implementation, and evaluation of assistive technology and digital technology for children with HIs to be included in this review. Refer to Textbox 1 for details regarding the inclusion and exclusion criteria. Studies were included if they focused on identifying, implementing, or evaluating assistive or digital technologies for children with HIs. Studies were limited to English-language, peer-reviewed journal studies published between January 2014 and September 2024. Studies of adults, nondigital technologies, or those focusing exclusively on signed language were excluded.

Textbox 1. **Inclusion and exclusion criteria.**

**Inclusion criteria:**

- Studies focused on hearing technology for children with hearing impairments (HIs).
- Studies involving digital technology, mobile apps, or computer-based interventions.
- Studies written in English.
- A study was only included in our review if the technology was specifically intended for language development outcomes such as vocabulary learning, grammar acquisition, speech production, or signed language comprehension.
- Digital tools used by children with HI may indirectly impact language development if they serve as communication aids or educational games. However, this review only included technologies mainly aimed at language learning or those directly measuring language-related outcomes.
- Studies published between 2014 and 2024 were included in this review. This period was chosen because mobile and app-based digital interventions have evolved rapidly since smartphones and tablets became widely used [26]. Earlier studies were largely based on technologies (eg, desktop-based or clinic-bound technologies such as computer-based training programs, CD-ROM–based tools, or fixed web platforms typically used in controlled settings) that are now quite different from the digital tools commonly used today (eg, app-based learning environments that support multimodal interaction and home-based language practice). From around 2014 onward, the growth of mobile learning technologies and app-based learning tools has had a substantial impact on digital environments for language learning, making this timeframe particularly relevant to the aims of our study.
- Journal studies.

**Exclusion criteria:**

- Studies were excluded from our review if the technology served primarily as a communication aid, accessibility tool, or general educational application without explicit language-learning objectives or language outcome measures.
- Abstract only, conferences and posters, study protocols without the published full-text study, and editorial letters.
- Studies exclusively focused on traditional sign-language instruction without a digital assistive learning component targeting broader communication, literacy, or educational participation outcomes were excluded.
- Studies focused on HIs in adults aged >18 years.
- Studies not written in English.
- Studies published before 2014.
- Nondigital technologies.

#### Screening and Selection Process

Eligible studies were identified using a 3-step process. In step 1, one reviewer (ADS) collected the search results and then removed duplicates. In step 2, two reviewers (ADS and AAM) reviewed the titles and abstracts of the identified studies that appeared to meet the inclusion criteria. In step 3, the same reviewers evaluated the full texts of the remaining publications that were identified in our searches to include potentially relevant publications. Covidence software (Veritas Health Innovation Ltd) was used to facilitate the screening, selection, and data management processes during the scoping review [25].

### Step 4: Data Extraction and Charting the Data

Data from the eligible studies were extracted using standardized data extraction forms developed by the research team (refer to Multimedia Appendix 1). Two separate data extraction tools were used to capture key details about digital technologies, including the type of technology, the communication preference (signed language or spoken language), app settings, app purpose (research-based or commercial), and app functionality (eg, language learning, math learning, literacy, or educational assistance). In addition, the extracted data included study characteristics such as the year of publication, the country in which the study was conducted, and the purpose of the study, as well as participants’ demographics, such as age, sex, and degree of HI. Outcome measures were also recorded to assess the impact of the technology.

### Step 5: Collating, Summarizing, and Reporting the Results

We grouped the studies based on their technological focus and summarized the key characteristics, including the study design, target populations, settings, research purposes, and reported limitations. We identified and outlined the features of the digital tools used for each group, together with the intended outcomes. This process was intended to highlight existing gaps and to inform future research or intervention designs.

## Results

### Overview of the Studies

**Figure 1.**
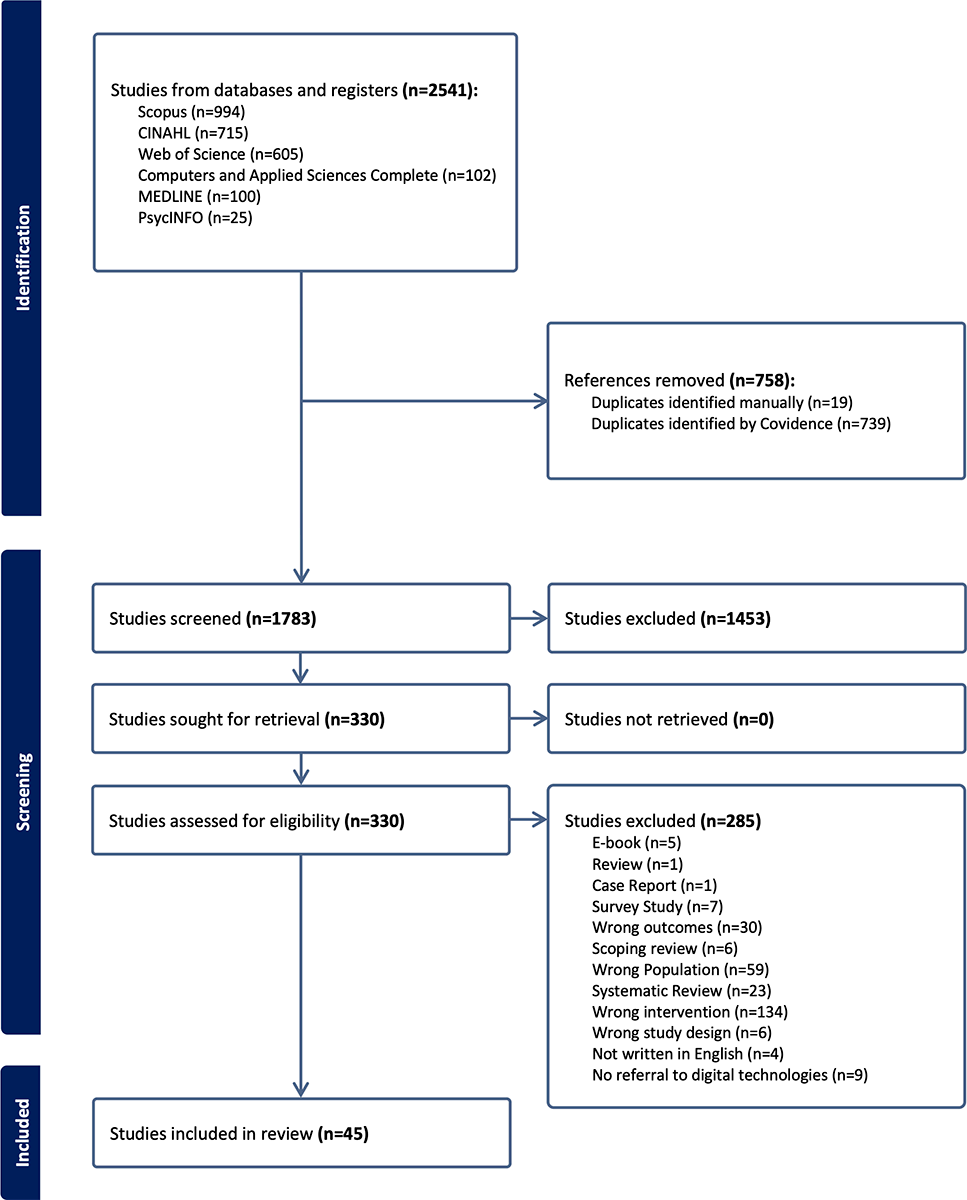
PRISMA (Preferred Reporting Items for Systematic Reviews and Meta-Analyses) flowchart for the selection of the included studies.

Initially, 2541 studies were imported into Covidence; after duplicate removal, 1783 citations were identified from electronic database searches and study references. After screening the titles and abstracts of these citations, 330 studies were included in the full-text review process. Of these 330 studies, 45 studies met the inclusion criteria, and 285 were excluded (refer to Figure 1). Data extraction was completed for the 45 studies that were considered eligible for this review. The PRISMA (Preferred Reporting Items for Systematic Reviews and Meta-Analyses) flowchart is shown in Figure 1.

**Figure 2.**
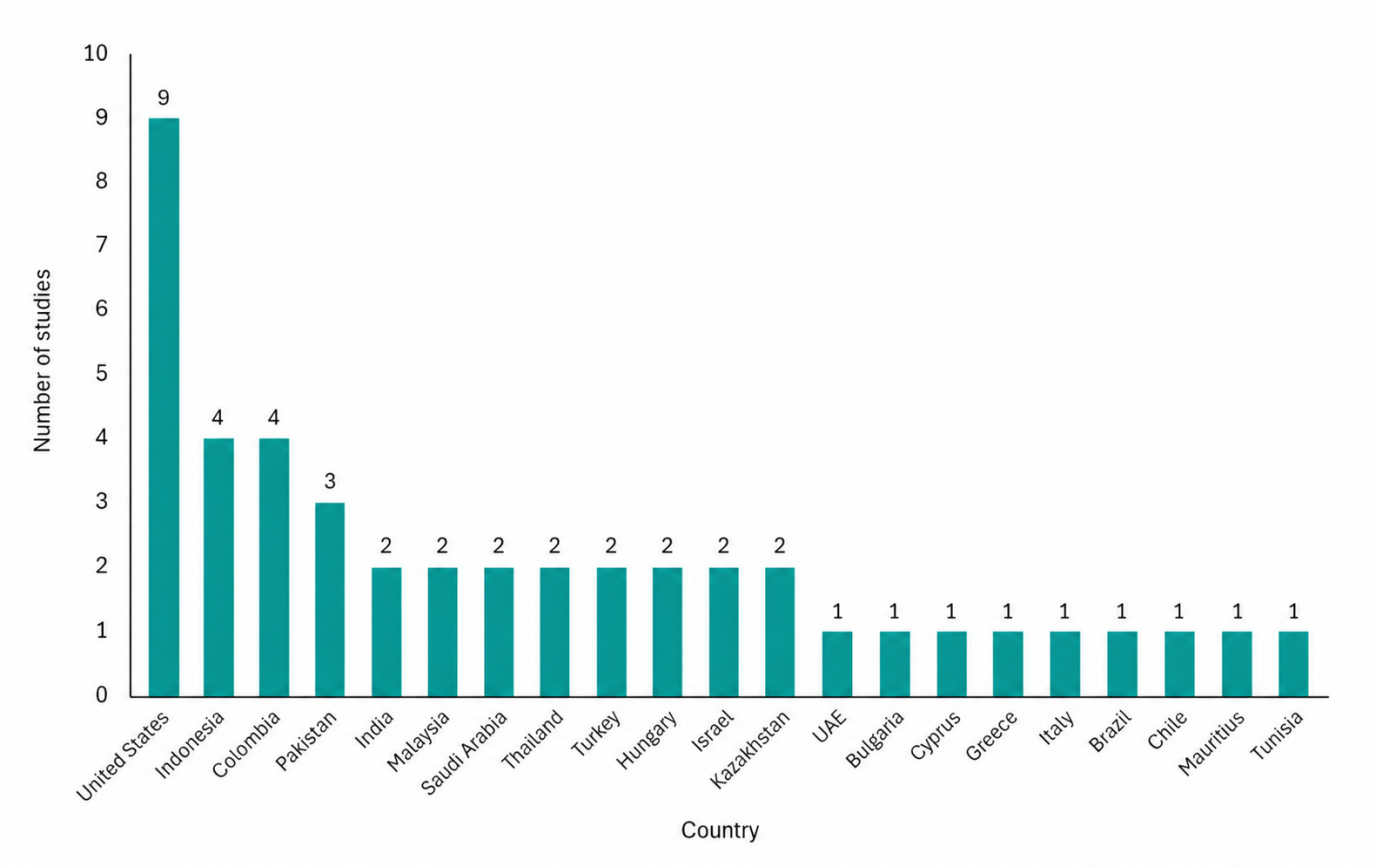
Distribution of included studies by country, showing the number of studies conducted across different geographical regions.

Figure 2 shows the geographical distribution of the included studies. Most studies were conducted in Asia (40%, 18/45), followed by Europe (22%, 10/45), and North America (20%, 9/45). All North American studies originated from the United States, which represented the largest national contribution. South America contributed 13% (6/45), primarily from Colombia (4/45), while Africa accounted for 4% (2/45), represented by Mauritius and Tunisia. Consistent with the population, concept, and context framework, geographical context (high-income vs low- and middle-income countries) is reported descriptively to contextualize access, scalability, and learning environments, rather than as a separate analytical focus.

Among the studies conducted in low‑ and middle‑income countries (n=24), Indonesia and Colombia contributed the highest number of studies (n=4 each), followed by Pakistan (n=3) (Table 2).

Of the 45 studies, only 18 studies involved parents and experts. Parents mainly supported progress monitoring [27-29] and were most engaged during the design stage [16,30-36]. A few studies also created dedicated interfaces for parents and experts [6,34], highlighting the role of technology in connecting home and professional contexts. In addition, testing was not limited to children, as parents and experts were also part of the evaluation process. Previous studies by Mood et al [30], Meinzen-Derr et al [37], and Shoaib et al [38] highlighted that technology requires the continuous input of parents, therapists, and teachers to maximize its impact.

**Table 2.** Distribution of studies across low- and middle-income countries (N=24).

Country	Value, n (%)
Colombia	4 (16.7)
Indonesia	4 (16.7)
Pakistan	3 (12.5)
India	2 (8.3)
Kazakhstan	2 (8.3)
Malaysia	2 (8.3)
Thailand	2 (8.3)
Turkey	2 (8.3)
Brazil	1 (4.2)
Mauritius	1 (4.2)
Tunisia	1 (4.2)
Total	24 (100)

### Participant Characteristics

The mean sample size across the included studies was relatively small, with an average of 23 participants. Participant ages ranged from 2 to 17 years, with a mean age of 10.6 years. The mean age of children was 11 years, and the mean sample size was 20 participants. Of the 45 included studies in this review, 27 reported participant gender, comprising 778 individuals. Of these individuals, 397 (51%) were male, and 381 (49%) were female. The remaining 18 studies (221 participants) did not specify gender information. All studies involved participants with HIs; however, only 21 studies provided details on the severity of hearing loss. In total, 8 studies reported profound hearing loss, 1 severe, 5 moderate, and 7 mild. The remaining 24 studies did not provide specific information regarding the extent of hearing loss. There was an inconsistency in the reporting of gender and demographic data; therefore, the aggregate data only reflect studies that reported this information.

### Types of Digital Technologies That Support Language Learning for Children With HIs

Five categories of digital technology were identified, namely mobile apps (n=26), digital books (n=6), extended reality, augmented reality, and virtual reality (n=5), computer-based programs (n=3), and other tools such as robots, tangible devices, or cross-platform systems (n=5; refer to Multimedia Appendix 1 and Table 3). Across the range of technologies, mobile apps consistently highlighted the importance of customization, interactivity, and visual reinforcement, whereas XR and tangible systems highlighted the importance of embodied and immersive approaches to learning.

**Table 3.** Digital technologies and features supporting spoken, signed, and bimodal language learning.

Purpose, type of technology, and key features	Frequency
Language learning, literacy, and math learning	
Mobile app	
Game-based approach: reward, level, and challenges.	4
Multimedia content: audio, video, images, text, animations, and colorful design.	8
Learning monitoring: activity log and progress report.	5
Interactive: real-time feedback, task manipulation, touch, speech, and gesture.	2
AI^a^ feature: optical character recognition, AI image classification, and object detection.	2
Online and offline functionality.	3
Real-time image processing	2
Parental support	2
Professional support	1
Vocabulary learning	2
Fingerspelling	2
Pronunciation, speech, and assistant	
Computer	
Game-based approach	3
Real-time feedback and visual feedback.	3
Learning progression	2
Education support and language learning	
AR^b^, MR^c^, and VR^d^	
Interactive object manipulation	4
3D avatar-based interaction	5
Visual learning	3
Spoken-language learning	
AAC^e^	
Symbol-based communication, speech output, and custom interface.	5
Language learning and education support	
Other (tangible or robot)	
Gestures, poses, speech synthesis, and signed-language tutoring; tablet-based display for videos, animations, and interactive exercises; multimodal engagement (auditory-verbal, visual, and cued speech approaches); and autonomous navigation and interaction with children in an adaptive learning environment, with real-time feedback and rewards.	3
Physical board games and tactile features.	3

a AI: artificial intelligence.

b AR: augmented reality.

c MR: mixed reality.

d VR: virtual reality.

e AAC: augmentative and alternative communication.

Various digital technologies have been developed to support language learning for children with HIs, each offering distinct features and learning approaches. Mobile-based apps, which were the most frequently represented, tended to incorporate diverse interactive elements, such as color, animations, and accessible font styles that were designed to engage young users [39,40]. Customization features appeared most frequently across all technology types, reflecting the need to accommodate variations in hearing levels and individual progress. Leveling systems were also suggested as essential additions, enabling content to be adapted according to the child’s stage of development [41,42]. XR and tangible technologies, such as robots, provided immersive and hands-on experiences, while computer-based and cross-platform software supported more structured or instructional content.

The learning goals that were supported by these technologies typically aligned with either spoken-language or signed-language development. Technologies that were designed to support spoken language, which were frequently delivered through mobile apps or AAC-based platforms, often focused on auditory memory and visual reinforcement [30,37]. These tools included speech-to-text, audio playback, activity logging, and real-time feedback to support receptive and expressive language skills. By contrast, technologies that supported signed-language learning relied heavily on visual modalities and incorporated elements such as video demonstrations of signs, fingerspelling, and articulation animations [43-45].

While mobile-based technologies dominated in frequency and feature integration, each type of technology had unique value depending on the targeted communication mode. Tools that supported spoken languages tended to integrate multimodal input (auditory and visual), whereas those supporting signed language were predominantly visual. These differences highlight the importance of aligning technological features with the sensory needs of children with HIs, as well as with their language preferences [31]. Customization, interactivity, and adaptability remain central to the effectiveness of these digital tools in diverse learning contexts.

### The Role of Digital Technology in Supporting Language Learning and Language-Mediated Academic Participation in Children With HIs

While this review focuses on language learning, many of the technologies described incorporate language development into more general learning activities, such as literacy, mathematics, science, and participation. As a result, these areas were also included whenever language comprehension, expression, or communication formed a core component of the learning process. Digital technologies served the following two main functions: (1) assistive and learning support (literacy, math, science, and classroom participation) and (2) language learning (spoken and signed languages).

### Assistive and Learning Support

Across the reviewed studies, assistive and learning support technologies for children with HIs consistently featured multimodal, interactive tools that adapted traditional curricula and presented them in more accessible and engaging formats. These technologies span various domains, including literacy, mathematics, science, and classroom participation, with an emphasis on personalized and inclusive learning experiences.

In terms of literacy, the most common tools used are digital storytelling and gamified apps to enhance reading comprehension, vocabulary acquisition, and phonemic awareness. Standard features included animated narratives, interactive feedback, and adaptive content, which not only maintained learner engagement, but also supported comprehension in both signed- and spoken-language contexts [46-52]. Several studies highlighted the role of e-books and storytelling apps in facilitating bilingual or bimodal literacy, reflecting a shift toward culturally and linguistically responsive learning materials.

In terms of math, digital tools often incorporate multimedia elements, such as visual aids, auditory prompts, and gesture-based input, to improve the understanding of mathematical concepts. A shared feature across these tools was their ability to break down abstract mathematical ideas into concrete, interactive tasks. Many platforms also integrated signed language or visual modeling to accommodate different learning styles [32,53-56]. These interventions indicated a common goal of making math education more inclusive and accessible, particularly by addressing language-related barriers that may impede conceptual understanding.

In terms of science and classroom learning, a variety of assistive technologies, such as AR apps and humanoid robots, were used to simulate real-world experiences to foster inquiry-based learning and promote social interaction [43,57-59]. Of note, some of these tools, such as humanoid robots, were designed for classroom use and to support parents and caregivers at home, thereby enabling them to reinforce language and academic skills in informal settings. This highlighted the increasing recognition of the home environment as a crucial context for learning, particularly when access to professional therapy was limited.

Differences among these categories reflected their learning objectives and sensory modalities. Literacy tools often focus on phonemic and vocabulary development through fingerspelling for signed languages and audiovisual storytelling for spoken languages [6,49]. Math tools prioritized logical reasoning and problem-solving through interactive formats [53,54,60], while science-oriented technologies were geared toward experiential learning and stimulating curiosity [61]. Across the domains, most of the tools incorporated multimodal input (visual, auditory, or gestural) and adaptive content features.

### Language Learning

A strong trend across spoken-language interventions for children with HIs was the increasing use of technology-enhanced tools that combined visual, auditory, and interactive elements to support diverse aspects of spoken-language development. These tools addressed various communication skills, ranging from auditory-verbal training and lip reading to speech articulation, phonemic awareness, and expressive language production.

One commonality among these interventions was the emphasis on multisensory input. Most apps integrated real-time visual and auditory feedback, allowing users to self-correct and reinforce their learning. For example, tools targeting articulation and auditory training often use game-based interfaces and visual prompts to help learners practice specific speech sounds and patterns in an engaging environment [5,27,33]. Similarly, lip-reading tools focused on visual speech cues, providing guided repetition and clear visual models to support speech recognition without relying on signed language [28,38].

AAC-based systems were another common strand, particularly for supporting expressive spoken language and pragmatic communication. These tools typically provided audiovisual pairings, customizable vocabularies, and structured sentence construction to support communication across different developmental stages [14,30,37,62]. Longitudinal research has also shown that AAC tools can be adapted to a child’s developing language abilities and can remain effective across contexts [63].

Despite these shared features, differences in focus and depth were noted. Some tools were narrowly designed for phoneme-level training or early auditory skills [27], while others provided comprehensive language systems with vocabulary expansion, grammar structuring, and social communication support [14,30]. The level of interaction also varied, ranging from passive repetition tasks to immersive, gamified environments with dynamic feedback loops [64].

Overall, these studies reflected a shift toward personalized, feedback-driven spoken-language learning, with tools tailored to different linguistic profiles, therapy goals, and developmental needs.

34

### Efficacy of the Tools for Language Learning

Table 4 summarizes the characteristics, interventions, and key findings of the 12 studies that reported efficacy outcomes. These studies covered spoken-language learning (AAC, phonemic awareness, and prosody) and signed-language learning, with 1 study also reporting academic outcomes. Where available, statistical results are reported descriptively to reflect the outcomes presented in the original studies, rather than to infer comparative effectiveness.

**Table 4.** Efficacy of digital technologies.

Study	Aim of the study	Name of the platform	Age of the children (years)	Key results
Mood et al [30]	To evaluate AAC^a^ on iPads for language development	AAC on iPad TALI^b^	3‐12	Over 24 weeks, children aged ≥5 years in the TALI^b^ group showed greater gains in pragmatic language (12.7-point increase in CELF-5^c^) vs a 6-point decrease in the TAU^d^ group (*P*=.04). For children under 5, the TALI group improved significantly in VABS^e^ Communication (86.7 to 99.1) and Social domains (91.8 to 97.4, *P*=.01), while the TAU group showed no significant change.
Meinzen-Derr et al [62]	To assess the TALI program’s impact on communication skills	TouchChat HD: AAC with WordPower (used in TALI)	3‐12	Over 24 weeks, children in the TALI group, compared to those in the TAU group, had significantly greater increases in the length of phrases they used to express themselves (*β*=.91 vs .15, respectively; *P*<.001). Similar ﬁndings were seen with conversational turn-taking and the number of different words spoken.
Meinzen-Derr et al [37]	To implement a language intervention that used AAC technology on iPad VR devices to enhance language development in children who are DHH^g^ with language underperformance	AAC software on iPad TouchChat HD	5‐10	^f^More than 24 weeks, median MLU^f^ increased from 2.41 (range 1.09-6.63; mean 2.88) to 3.68 (range 1.97-6.81; mean 3.62; *P*=.002). Median total words were 251 (range 101-458) and median different words were 100 (range 36-100) at baseline, both increasing significantly over time. Total words: *β*=26.8 (7.1); *P*=.001 and different words: *β*=8.0 (2.7); *P*=.008. Mean (SD) values increased from 3.15 (2.09) to 3.86 (1.81) for MLU, from 265.4 (130.1) to 357.8 (159.2) for total words, and from 96.6 (44.2) to 125.2 (42.8) for different words; mean turn length showed minimal change.
Meinzen-Derr et al [14]	To determine whether integrating AAC core-word language strategies improves speech-language outcomes in young children who are deaf or hard of hearing	AAC strategies using TouchChat HD with WordPower by Silver Kite	4-11	The study reported high satisfaction with AAC strategies and notable language gains: MLU increased by 38%, NDW by 39%, and the Pragmatic Checklist Score rose from 68.8 to 95.5 (*P*=.01).
Mohammad et al [34]	To test a game-based mobile app for a signed language	My Sign	6‐12	The study showed that children using the mobile app scored significantly higher in the post-quiz, with a 60% improvement in Group A. The *t* test (*P*=.01) confirmed the app’s positive impact, and the Eta squared (0.585) indicated a strong effect size. The game-based learning approach led to higher engagement and better learning outcomes compared to the traditional method.
Ployjiw and Michel [35]	To use the AR^h^ book to teach vocabulary and signs	AR book	5‐9	The results showed that the efficiency of the lesson in the pretest and the exercise during the study (E1)^i^ averaged 85.33%, while the posttest (E2) averaged 87%.
Sztahó et al [65]	To improve prosody using CAPT^j^	CAPT	6‐10	The CAPT app significantly improved prosody in children with HIs (*t*_14_=4.07; *P*<.001), with strong agreement between teachers’ and automatic scores (*r*=0.87). Gains were observed across all hearing levels, particularly among children with mild hearing loss.
Vasel and Ragonis [32]	To assess multimedia impact on academic outcomes	Multimedia instruction	7‐11	The program significantly improved students’ language and math skills, with gains in semantics, reading (49.5→60.5), grammar (63.3→66.9; *P*=.001), and math mapping. Morphology showed slight improvement; syntax remained unchanged.
Lawal et al [39]	To evaluate English learning via a digital app	A mobile app named ‘‘Hausar Kurma’’	5-7	The study showed that the mobile app significantly improved English learning among Hausa-speaking students with hearing impairment (pretest median = 30; posttest median=90; *z*=2.82; *P*<.05; *r*=0.633; binomial *P*=.01).
Techaraungrong et al [55]	Math learning (counting, addition, and subtraction) with language components through a signed language	Multimedia app for arithmetic learning	7	The study revealed that multimedia-based learning significantly outperformed traditional instruction (*z*=4.545; *P*<.001), improving counting, addition, and subtraction. Students reported better motivation, understanding, and independence, supporting multimedia’s effectiveness and the need for inclusive design.
Alsalem and Alzahrani [46]	Language learning (phonemic awareness, vocabulary, writing, and reading comprehension)	Custom-designed e-books	10‐12	There were statistically significant between-group differences across all outcome measures, with the experimental group demonstrating superior performance in phonemic awareness (*t*_39_=11.25; *P*<.001; *d*=1.77), writing (*t*_39_=12.18; *P*<.001; *d*=2.03), vocabulary (*t*_39_=7.25; *P*<.001; *d*=1.15), and reading comprehension (*t*_39_=6.29; *P*<.001; *d*=0.99). All observed effects were large.
Joy et al [31]	To develop and evaluate a mobile-based tool for learning sign vocabulary to improve deaf and Deaf children’s language acquisition.	SiLearn	School-age children	The SiLearn program significantly improved vocabulary, with higher gains in the experimental group (mean 21.43, SD 7.45) vs the control group (mean 11.07; *t*_22.91_=2.926; *P*=.003), confirming its effectiveness.

a AAC: augmentative and alternative communication.

b TALI: Technology-Assisted Language Intervention.

c CELF-5: Clinical Evaluation of Language Fundamentals, Fifth Edition.

d TAU: treatment as usual.

e VABS: Vineland Adaptive Behavior Scale.

f MLU: mean length of utterance.

g DHH: deaf and hard of hearing.

h AR: augmented reality.

i E: efficiency index.

j CAPT: computer-aided pronunciation training.

Across the 5 AAC-focused studies, 4 studies [14,30,37,62] reported positive efficacy outcomes in specific language domains, including vocabulary growth, increased utterance length, and improvements in pragmatic communication, while 1 study did not include relevant efficacy data. For instance, in a 24-week study using AAC on iPads, the mean length of utterance increased from 2.41 to 3.68 (*P*=.002), the total words increased by 26.8 (*P*=.001), and different words increased by 8 (*P*=.008) [30]. In the randomized controlled trial of the Technology-Assisted Language Intervention (TALI), children aged ≥5 years showed a 12.7-point increase in Clinical Evaluation of Language Fundamentals, Fifth Edition Pragmatics Profile scores compared to a 6-point decrease in the treatment-as-usual group (*P*=.04) [62]. For children aged <5 years, significant improvements were observed in the Vineland Adaptive Behavior Scale (VABS) Communication (86.7-99.1) and social domain scores (91.8-97.4; *P*=.01). In addition, children in the TALI group demonstrated greater gains in the number of different words spoken (82-104) compared to the treatment-as-usual group, which increased from 82 to 87 [14]. This difference was statistically significant (*β*=11.04 vs *β*=2.65; *P*=.007), further emphasizing the more substantial impact of the TALI approach on language development [62]. Preliminary results suggest promise; however, most research designs had small sample sizes, short intervention durations, and heterogeneous outcome measures.

Only 12 studies reported efficacy outcomes; however, they had small sample sizes, short intervention periods, and methodological variability. Thus, the findings should be interpreted as exploratory rather than confirmatory. One study reported on the effectiveness of computer-assisted pronunciation training (CAPT) in supporting speech therapy for children with HIs [65]. Sztahó et al [65] found that, after a 3-month intervention, children using the CAPT app showed significantly improved prosody compared to the control group, with strong agreement between automatic assessments and teachers’ ratings. Improvements were most notable in children with mild hearing loss, but were present across all levels [46]. Students in the treatment group showed large improvements in phonemic awareness, writing, vocabulary, and reading (all *P*<.05) with large effect sizes (*d*=0.99‐2.03) [46].

Several studies (n=5) demonstrated the effectiveness of digital tools in enhancing the acquisition and development of signed languages [31,34,35,39,55]. A mobile app significantly improved signed-language learning compared to traditional methods (*P*=.01; η^2^=0.585) because of its engaging, game-based features [34]. Ployjiw et al [35] reported vocabulary gains using AR books, with scores increasing from 7.20 to 8.70 (*P*=.01) and instructional efficiency exceeding 85%. High satisfaction was noted, particularly with signed-language videos and parental involvement. The SiLearn app (developed by Joy et al [31]) also showed significant vocabulary improvements, confirming its positive impact. Another study [39] reported a sharp increase in English scores, while Techaraungrong et al [55] found that multimedia instruction outperformed traditional methods in basic math by enhancing motivation and independent learning. One study [32] reported on the efficacy of digital tools in supporting academic outcomes such as language, math, and classroom learning for children with HIs. Significant gains were observed in reading, grammar, and math mapping. Refer to Table 4 for the full results. Although several studies showed statistical significance, the limited number of studies on the efficacy of digital technology makes it difficult to draw conclusions on the comparative effectiveness of the intervention.

## Discussion

### Principal Findings

In this scoping review, we synthesized evidence regarding how digital technologies were used to support language learning for children with HIs across home, school, and clinical contexts. Of these technologies, mobile apps emerged as the most frequently used tools due to their accessibility, ease of use, and adaptability to different contexts [34,66]. This suggests that the ease of deployment is prioritized over the pedagogical depth and the mechanisms of language learning.

By contrast, other technologies, such as computer-based programs, AR, VR, tangible user interfaces, and social robotics, were implemented less frequently, probably due to higher costs, greater complexity, or limited availability [57,59,67]. The scoping review findings reveal 3 structural patterns in the existing evidence base, which affect the way in which children with HIs acquire languages through the use of digital technologies. These patterns relate to the dominance of mobile technologies, the relative focus on the use of signed-language vs spoken-language interventions, and the use of digital technologies in the classroom, the clinic, and the home.

One of the major findings of this review is that the majority of the mobile apps were created for signed-language learning, with a strong emphasis on enabling children to develop their communication skills through visual language [57,68]. This is also understandable since the relative ease of designing signed-language technologies may also be a factor, since it is much more easily implemented in mobile apps without the need for any complex auditory-processing capabilities. Besides the purpose of signed-language learning, other apps included speech-to-text functionality for general purposes of communication, as well as a variety of assistive learning apps such as literacy, reading, mathematics, and classroom apps [31,35,49].

By contrast, apps focusing on the development of spoken language were relatively limited, particularly for younger children. Existing spoken-language tools typically supported auditory training for children who used hearing aids or cochlear implants by using phoneme recognition, AAC, and interactive exercises to enhance speech, literacy, and pragmatic communication [27,30,48]. While the number of spoken-language apps was relatively small, both types of apps, whether focused on signed or spoken language, shared the same goal of helping children with HIs to communicate more effectively and strengthen their communication skills [30,32]. Despite this imbalance, both sign- and speech-oriented tools shared the overarching goal of improving children’s ability to communicate effectively, which is essential for academic achievement, social inclusion, and overall quality of life [69].

There were also a number of studies (n=10) related to the design and development of spoken-language apps that were targeted at young children, which may indicate that there is a relative lack of digital interventions in this area. This has significant analytical connotations, as it has been demonstrated that early childhood is an important period in the development of spoken language, where input is maximally effective [70,71]. There may be several reasons why there is a relative lack of early childhood spoken language tools, including the developmental sensitivity, complexity, and therapy requirements to support early spoken-language development in children with HIs, rather than later ages or signed-language development. Nevertheless, digital interventions for early spoken-language development have the potential to serve as effective and low-cost tools to support therapy in contexts where access to professional support is limited. It is therefore essential to prioritize the development of the body of research on tools to support spoken-language development in association with family-based interventions and therapy in early childhood, as this is the period where interventions are maximally likely to have long-term effects.

An important finding of this study highlighted the value of designing mobile apps that were both interactive and engaging, particularly for children with HIs. Features such as games, modular activities, and visual feedback helped to maintain attention and to support learning [34]. This aligned with theories of multimodal learning, which suggest that combining auditory, visual, and kinesthetic inputs enhances comprehension and retention [72]. Evidence has shown that multimedia-based approaches demonstrated a stronger and more consistent impact on improving math skills compared to conventional teaching methods, as multimedia-based approaches enhanced students’ motivation, improved comprehension, and promoted greater independence in learning [55]. Nevertheless, visual design must also take users’ comfort and cultural relevance into account. Prolonged screen time may make children with HIs sensitive to harsh colors, such as bright red, which can cause eye strain [35]. In addition, culturally relevant content using familiar visuals, language, and contexts can improve engagement and comprehension [73]. Therefore, while rich interactive features enhance learning, they should be balanced with thoughtful visual and cultural design to ensure accessibility, comfort, and meaningful engagement.

We observed that most of the digital apps remained classroom-oriented and were rarely extended to a therapeutic setting or to home-based practice. With regard to spoken languages, multisensory approaches combining auditory and visual feedback in phoneme training, AAC platforms, or gamified exercises were common. This approach aligned with evidence showing that pairing auditory training with visual reinforcement strengthened outcomes [74]. Signed-language apps typically emphasized vocabulary building and visual communication, but often relied on drill-based practice, with limited support for family engagement or integration into daily social and classroom interactions. Overall, many of the tools lacked progression, therapist dashboards, and parent-engagement features. Reviews of parent-mediated interventions have raised similar concerns, noting that family involvement and asynchronous support were often underused [75]. This highlights a gap in tools that actively involve families and provide opportunities for sustained, adaptive practice at home.

Most of the available evidence comes from high-income countries. Only a handful of studies examined interventions in low- and middle-income countries, despite these being the settings in which access to therapists and structured educational services is most limited. In these contexts, home-based practice and community-led learning frequently represent the primary pathways for children’s communicative development. Addressing this challenge requires adapting and evaluating digital tools in resource-limited settings to ensure that they are relevant, sustainable, and responsive to the needs of both spoken- and signed-language users. Future work should therefore prioritize large-scale trials, long-term follow-up, and the cultural adaptation of digital interventions to ensure sustainability and broader impacts.

In terms of efficacy, AAC interventions demonstrated the strongest and most consistent evidence by improving utterance length, vocabulary, and pragmatic communication [14,37,62]. Signed-language apps, while effective for vocabulary and visual communication, often produced narrower outcomes that were limited to word-level learning [31,76]. Academic-oriented digital tools showed broader benefits in improving reading, writing, and mathematics, although their focus was less specifically on communication outcomes. Furthermore, CAPT interventions provided promising evidence for speech training and prosody, but the findings remain preliminary and were based on small-scale studies. Despite the positive outcomes, the evidence base remains limited. Many studies were short-term, small-scale, or lacked rigorous statistical testing, leaving questions about long-term efficacy and generalizability unanswered [13]. Few tools integrated therapist dashboards, adaptive progression, or structured parental involvement; these features could extend the benefits beyond drill-based practice. This review contributes to the field by providing an understanding of the role of current digital technologies in the facilitation of language learning across spoken-, signed-, and bimodal-language modalities, including the gaps in early spoken-language interventions, parent-mediated interventions, and culturally scalable interventions.

### Limitations

This scoping review has several limitations. First, the search was limited to studies published in English, which may have led to the exclusion of relevant studies published in other languages. As a result, the findings may not fully represent global perspectives, particularly in non-English-speaking regions in which important work in this area may take place. Second, while efforts were made to include studies from reputable sources, some relevant interventions were reported in lower-impact journals, which may have affected the overall quality and reliability of the included evidence. Third, restricting the search to studies published after 2014 may have excluded earlier work on computer-assisted language-learning technologies. Nevertheless, the aim was to synthesize evidence relevant to contemporary digital learning environments that are more relevant to current educational and clinical practice. As this study is a scoping review, the purpose was not to evaluate the quality of the included studies; therefore, the results should not be considered indicative of the effectiveness of the interventions. Additionally, the results may have been influenced by publication bias, selective results reporting, and the exclusion of non-English publications, which may have contributed to the lack of representation of certain regions. Moreover, the development of technology may affect the longevity of the tools identified in the study.

### Conclusion

This scoping review was intended to synthesize the existing literature concerning the use of digital technology to promote the development of languages among children with HIs in different settings such as the home, school, and clinic. The existing literature concerning the use of digital technology to promote language development among children with HIs indicates that there are 3 patterns that can be used to explain the use of digital technology to promote language development among children with HIs.

Mobile technology was the most widely used digital approach to promote language development among children with HIs due to its accessibility and adaptability across contexts. Among the mobile technologies, the use of signed-language interventions was the most commonly used to promote language development among children with HIs. The use of signed languages was effective in promoting vocabulary development among children with HIs. However, there was no existing literature concerning language development other than vocabulary among children with HIs. On the other hand, spoken-language interventions were less commonly used to promote language development among children with HIs. However, the existing literature concerning the use of AAC was effective in promoting vocabulary and language development among children with HIs in structured contexts and with the assistance of therapists and parents.

Another major gap that we identified is the early development of spoken language. Although it is acknowledged that the period of early childhood is a critical period for the development of language, there are still very few digital interventions for the development of spoken-language skills for young children with HIs. In addition, the interventions that were implemented did not have the essential characteristics of being adaptable, having therapist dashboards, and involving parents.

In conclusion, although digital technology is acknowledged as a potential facilitator of language development for children with HIs, this potential is limited by the imbalance between the 2 modalities and fragmented interventions. Further studies should aim at the co-design and coevaluation of digital technology for the development of both signed and spoken languages for children with HIs, as well as the facilitation of the continuation of language learning for children both at home and school.

## Supplementary material

10.2196/85066 Multimedia Appendix 1 Summary of the included studies.

10.2196/85066 Checklist 1 PRISMA ScR checklist.

## Abbreviations

AAC: augmentative and alternative communication
AR: augmented reality
CAPT: computer-assisted pronunciation training
HI: hearing impairment
PRISMA: Preferred Reporting Items for Systematic Reviews and Meta-Analysis
PRISMA-ScR: Preferred Reporting Items for Systematic Reviews and Meta-Analysis Extension for Scoping Reviews
RQ: research question
TALI: Technology-Assisted Language Intervention
VR: virtual reality
XR: extended reality
