# Supplementary material for: Digital Technologies for Children With Hearing Impairments to Support Language Learning: Scoping Review
Source: JMIR Rehabil Assist Technol. 2026 Jun 16;13:e85066. doi: 10.2196/85066 (PMC13272877; doi:10.2196/85066)
Supplement: Multimedia Appendix 1 [file rehab-v13-e85066-s001.pdf]

Digital Technologies for Children With Hearing Impairments to Support Language Learning: Scoping Review

**Author**	**Country**	**Setting**	**Tech Type**	**Name of the platform**	**Aim**	**Learning Approach**	**Age of the children**
(Joy et al., 2020)	India	Mixed	Mobile app with video-based sign dictionary and optical character recognition (OCR)	SignDict	Sign Language Learning	Vocabulary Learning (ISL)	12.4 years
(Lawal et al., 2024)	Cyprus	School	Mobile Apps Android	Hausar Kurma	Sign Language Learning	Alphabet Learning (English Hausa Sign Language)	5-7 years
(Imashev et al., 2024)	Kazakhstan	Mixed	Mixed Reality (HoloLens 2)	Mixed Reality Application	Sign Language Learning	Vocabulary Learning	9-14 years
(Alnasif et al., 2022)	Saudi Arabia	School	Mobile Apps with real-time image processing and educational Technology	Mushir	Sign Language Learning	Basic Concept Learning (Arabic Sign Language): Letters, Shapes, Numbers, and Colours	4-7 years
(Joy et al., 2019)	India	Mixed	Mobile Apps-uses AI (Deep Learning and OCR)	SiLearn	Sign Language Learning	Vocabulary Learning	School-aged children

Digital Technologies for Children With Hearing Impairments to Support Language Learning: Scoping Review

Author	Country	Setting	Tech Type	Name of the platform	Aim	Learning Approach	Age of the children
(Beisov et al., 2024)	Kazakhstan	School	Computer-based Gesture recognition using AI and CNNs	Kazakh Gesture Recognition System	Sign Language Learning	Alphabet Learning (Kazakh Sign Language)	Not specified
(Mohammad et al., 2022)	UAE	School	Mobile Apps (Android)- learn sign language	MySign	Spoken Language Learning	Concept and Vocabulary Learning (ArSL)	7–11 years
(Firdous et al., 2019)	Pakistan	Therapy	Mobile Apps (Android)- Receptive language vocab spoken	Talking kids	Spoken Language Learning	Receptive Vocabulary Learning (Animals, Fruits, Colors, Relationships, Daily Routine Items)	2-6 years
(Shoaib et al., 2018)	Pakistan	Mixed	Software application for laptops.	LOSINA (Learning Application without Sign Language for Profound Hearing-Impaired Children)	Spoken Language Learning	World Articulation Training (Lip Reading Based)	5-8 years
(Dayawati et al., 2016)	Indonesia	NA	Mobile Apps (Android): lip reading	A-TooLips (Advanced TooLips)	Sign and Spoken Language Learning	Speech Production and Vocabulary Learning (Lip Reading-based)	4-7 years

Digital Technologies for Children With Hearing Impairments to Support Language Learning: Scoping Review

**Author**	**Country**	**Setting**	**Tech Type**	**Name of the platform**	**Aim**	**Learning Approach**	**Age of the children**
(Navarro-Newball et al., 2014)	Colombia	Therapy	Video game with speech recognition technology.	Talking to Teo	Spoken Language Learning	Speech Therapy	4-12 years
(S. Cano et al., 2018)	Colombia	Therapy	Games on PC and tablet platforms	Talking to TEO, Pre-lingua, and Vivoso	Spoken Language Learning	Speech Therapy	5-11 years
(Meinzen-Derr et al., 2021)	USA	Mixed: Home, and Therapy	AAC software on iPads (iOS)	Touch Chat HD - AAC with WordPower (used in TALI)	Spoken Language Learning	AAC	5-8 years
(Meinzen-Derr et al., 2019)	USA	Mixed: Home, and Therapy	Mobile apps (iPad) (AAC)	Touch Chat HD with WordPower	Spoken Language Learning	AAC	3.11-10.8 years
(Meinzen-Derr et al., 2017)	USA	Mixed: Home, and Therapy	Mobile app for iPads (AAC)	TouchChat HD WordPower	Spoken Language Learning	AAC	5-10 years
(Mood et al., 2022)	USA	Mixed: Home, and Therapy	Mobile app for iPads (AAC)	TALI (Technology-Assisted Language Intervention)	Spoken Language Learning	AAC	3-12 years

Digital Technologies for Children With Hearing Impairments to Support Language Learning: Scoping Review

**Author**	**Country**	**Setting**	**Tech Type**	**Name of the platform**	**Aim**	**Learning Approach**	**Age of the children**
(King et al., 2020)	USA	Mixed: Home, and Therapy	Mobile app for iPads (AAC)	Various AAC systems, including Proloquo2Go and Dynavox Gateway	Spoken Language Learning	AAC	2-15 years
(Rocha et al., 2023)	Brazil	School	Tangible assistive technology	My Little Sign	Assistive and Learning Support	Alphabet and Vocabulary Learning (Portuguese and the Brazilian Sign Language)	7-10 years
(Sandra Cano et al., 2018)	Colombia	Mixed	Tangible technologies integrated with IoT (RFID, Arduino, Bluetooth)	Phonomagic, Caseto	Assistive and Learning Support	Vocabulary and Music Learning (Auditory)	7-11 years
(Ployjiw & Michel, 2023)	Thailand	School	Augmented Reality (AR)	AR-Book	Assistive and Learning Support	Vocabulary Learning	6-7 years
(Chan et al., 2022)	French (Mauritius)	School	Gamification through desktop and mobile-based tools	NA	Learning Support	Gamification and Literacy	8-12 years

Digital Technologies for Children With Hearing Impairments to Support Language Learning: Scoping Review

Author	Country	Setting	Tech Type	Name of the platform	Aim	Learning Approach	Age of the children
(Alsalem & Alzahrani, 2024)	Saudi Arabia	Mixed	E-books (interactive and digital).	NA	Assistive	Literacy Learning	10-12 years
(Zainuddin et al., 2022)	Malaysia	School	Augmented Reality (AR)	PekAR-Mikroorganisma	Assistive and Learning Support	Science Learning	12-13 years
(Mohamad & Hashim, 2021)	Malaysia	School	Mobile Apps (Android)	KoTBaM and Learning Fakih	Assistive and Learning Support	Literacy learning	7-12 years
(Sztahó et al., 2018)	Hungary	School	Computer-based application for teaching prosody	CAPT	Assistive and Learning Support	Prosody Learning (intonation, rhythm, accent)	8-14 years
(Czap et al., 2019)	Hungary	School	Computer-based application using multimodal visualizations and 3D modelling (Speech Assistant)	Speech Assistant (SA)	Assistive and Learning Support	Speech Production	8-14 years

Digital Technologies for Children With Hearing Impairments to Support Language Learning: Scoping Review

**Author**	**Country**	**Setting**	**Tech Type**	**Name of the platform**	**Aim**	**Learning Approach**	**Age of the children**
(Lekova et al., 2021)	Bulgaria	Home	Socially assistive robot (SAR)	Pepper (robot-assisted system)	Assistive and Learning Support	Auditory and Visual Stimulation	Infant toddler, N/A
(Goker et al., 2016)	Turkey	School	Mobile Apps (Educational software for PC)	NA	Assistive and Learning Support	Concept Learning	4-7 years
(Herzig & Allen, 2023)	USA	NA	Mobile App (iOS)	The Baobab	Assistive and Learning Support	Storytelling	5-8 years
(Flórez-Aristizábal et al., 2019)	Colombia	NA	Tangible cards and a desktop-based digital storytelling application.	NA	Assistive and Learning Support	Storytelling	9-14 years
(Brouwer et al., **2017**)	USA	Mixed	Mobile Apps iOS (Apple iPads)	Endless Alphabet	Assistive and Learning Support	Vocabulary Learning	3,11-4,3 years
(Yaman et al., 2016)	Turkey	Mixed	Mobile apps (Story Book)	Storybook and Story Map	Assistive and Learning Support	Reading and Literacy	9-13 years

Digital Technologies for Children With Hearing Impairments to Support Language Learning: Scoping Review

**Author**	**Country**	**Setting**	**Tech Type**	**Name of the platform**	**Aim**	**Learning Approach**	**Age of the children**
(Majorano et al., 2024)	Italy	Therapy	Computer-based program	Simo-Syl Program	Assistive and Learning Support	Literacy	Approximately 5 years
(Véliz et al., 2017)	Chile	School	Multimodal digital books for desktop use.	NA	Assistive and Learning Support	Reading Comprehensive	4- 14 years
(DeForte et al., 2020)	USA	Mixed	Mobile Apps - Digital Story Book (iOS)	Hear Me Read	Assistive and Learning Support	Literacy	2-12 years
(Bouzid et al., 2016)	Tunisia	School	Mobile educational game 3D Avatar	MemoSign	Assistive and Learning Support	Sign writing and Vocabulary	9-16 years
(Eden, 2014)	Israel	School	Computer-based VR software	NA	Assistive and Learning Support	Storytelling	4-7 years
(Hanafi et al., 2019)	Indonesia	NA	Mobile Apps (Android) Quran reading	Qur'ani	Assistive and Learning Support	Reading Fluency	15-18 years

Digital Technologies for Children With Hearing Impairments to Support Language Learning: Scoping Review

**Author**	**Country**	**Setting**	**Tech Type**	**Name of the platform**	**Aim**	**Learning Approach**	**Age of the children**
(Suarsana, 2021)	Indonesia	School	Digital Book	Interactive Digital Mathematics Book	Learning Support	Learning Math	12-14 years or 13 -15 years
(Vasel & Ragonis, 2024)	Israel	NA	Integrated technology-based mobile learning with laptops and tablet applications	NA: described as 21 language apps and 17 math apps	Learning Support	Learning Math	9-12 years
(Parvez et al., 2019)	Pakistan	NA	Mobile Apps (Android)	NA: mobile application for PSL-based math learning	Learning Support	Learning Math	5-10 years
(Shelton & Parlin, 2016)	United States	Mixed	Digital Mathematics Book	GeePerS*Math	Learning Support	Learning Math	9-12 years
(Techaraungrong et al., 2017)	Thailand	NA	Mobile Apps (Multimedia Application): arithmetic	NA	Learning Support	Learning Math	7 years
(Harisman et al., 2023)	Indonesia	School	Mobile Apps - Tangible (e-comics via Pixton)	Proton-Electron Math E-Comic	Learning Support	Learning Math	7-15 years

Reference

Alnasif, H., Alyahya, L. A., Alromaih, H., Alhelal, G., Barnawi, N., Altamim, A., & Albassam, S. A. A. (2022). Mushir: An Arabic Edutainment Application For Deaf and Hard of Hearing Children Using Real-time Image Processing. URL: http://paper.ijcsns.org/07_book/202209/20220983.pdf [Accessed 2026-05-26]

Alsalem, M. A., & Alzahrani, H. A. (2024). In search of language development for students who are hard of hearing: measuring the effectiveness of assistive technologies through teaching practices. *Assistive Technology*, *36*(1), 3-10. [doi: 10.1080/10400435.2023.2193761] [Medline: 36996032]

Beisov, N., Madyarova, G., & Kerimbayev, N. (2024). Gesture recognition technology: a new dimension in human-computer interaction interface. *Indonesian Journal of Electrical Engineering and Computer Science*, *35*(2), 1311-1324. https://doi.org/10.11591/ijeecs.v35.i2.pp1311-1324

Bouzid, Y., Khenissi, M. A., Essalmi, F., & Jemni, M. (2016). Using educational games for sign language learning - A signwriting learning game: Case study. *Educational Technology and Society*, *19*(1), 129-141. https://www.scopus.com/inward/record.uri?eid=2-s2.0-84957664346&partnerID=40&md5=2a45f6553ac38283d2306d153dfe1896

Brouwer, K., Downing, H., Westhoff, S., Wait, R., Entwisle, L. K., Messersmith, J. J., & Hanson, E. K. (2017). Effects of Clinician-Guided Emergent Literacy Intervention Using Interactive Tablet Technology for Preschool Children With Cochlear Implants. *Communication Disorders Quarterly*, *38*(4), 195-205. https://doi.org/10.1177/1525740116666040

Cano, S., Collazos, C. A., Flórez Aristizábal, L., Gonzalez, C. S., & Moreira, F. (2018). Towards a methodology for user experience assessment of serious games with children with cochlear implants. *Telematics and Informatics*, *35*(4), 993-1004. https://doi.org/10.1016/j.tele.2017.09.011

Cano, S., Flórez-Aristizábal, L., Collazos, C. A., Fardaoun, H. M., & Alghazzawi, D. M. (2018). Designing interactive experiences for children with cochlear implant. *Sensors*, *18*(7), 2154.

Chan, G. L., Santally, M. I., & Whitehead, J. (2022). Gamification as technology enabler in SEN and DHH education. *Education and Information technologies*, *27*(7), 9031-9064. https://doi.org/https://dx.doi.org/10.1007/s10639-022-10984-y

Czap, L., Pintér, J. M., & Baksa-Varga, E. (2019). Features and results of a speech improvement experiment on hard of hearing children [Article]. *Speech Communication*, *106*, 7-20. https://doi.org/10.1016/j.specom.2018.11.003

Dayawati, R. N., Sulistiyo, M. D., Rani, M., Nistia, R. M., Linda, D. N., & Suwarsono, L. W. (2016). The implementation of A-TooLips, a learning mobile application for deaf children to produce words. *Jurnal Teknologi*, *78*(5), 405-411. https://doi.org/10.11113/jt.v78.8345

DeForte, S., Sezgin, E., Huefner, J., Lucius, S., Luna, J., Satyapriya, A. A., & Malhotra, P. (2020). Usability of a Mobile app for improving literacy in children with hearing impairment: focus group study. *JMIR human factors*, *7*(2), e16310. [doi: 10.2196/16310] [Medline:

32205305]

Eden, S. (2014). Virtual intervention to improve storytelling ability among deaf and hard-of-hearing children. *European Journal of Special Needs Education*, *29*(3), 370-386. https://doi.org/10.1080/08856257.2014.909177

Firdous, S., Wahid, M., Ud Din, A., Bakht, K., Khan, M. Y. A., Batool, R., & Noreen, M. (2019). Android based receptive language tracking tool for toddlers. *International Journal of Advanced Computer Science and Applications*, *10*(3), 589-595. https://doi.org/10.14569/IJACSA.2019.0100375

Flórez-Aristizábal, L., Cano, S., Collazos, C. A., Benavides, F., Moreira, F., & Fardoun, H. M. (2019). Digital transformation to support literacy teaching to deaf Children: From storytelling to digital interactive storytelling. *Telematics and Informatics*, *38*, 87-99. doi: 10. 1145/3290605.3300240]

Goker, H., Ozaydin, L., & Tekedere, H. (2016). The effectiveness and usability of the educational software on concept education for young children with Impaired Hearing. *Eurasia Journal of Mathematics, Science and Technology Education*, *12*(1), 109-124. https://doi.org/10.12973/eurasia.2016.1207a

Hanafi, Y., Hendrawan, H. J., & Hakim, I. N. (2019). Accelerating Qurán reading fluency through learning using QURáNI application for students with hearing impairments. *International Journal of Emerging Technologies in Learning*, *14*(6), 110-132. https://doi.org/10.3991/ijet.v14i06.9863

Harisman, Y., Dwina, F., Nasution, M. L., Amiruddin, M. H., & Syaputra, H. (2023). THE DEVELOPMENT OF PROTON-ELECTRON MATH E-COMIC TO IMPROVE SPECIAL NEEDS STUDENTS' MATHEMATICAL CONCEPTS UNDERSTANDING. *Infinity Journal*, *12*(2), 359-376. https://doi.org/10.22460/infinity.v12i2.p359-376

Herzig, M., & Allen, T. E. (2023). Deaf Children's Engagement with American Sign Language-English Bilingual Storybook Apps. *Journal of Deaf Studies & Deaf Education*, *28*(1), 53-67. https://doi.org/10.1093/deafed/enac032

Imashev, A., Kydyrbekova, A., Oralbayeva, N., Kenzhekhan, A., & Sandygulova, A. (2024). Learning sign language with mixed reality applications - the exploratory case study with deaf students. *Education and Information technologies*. https://doi.org/10.1007/s10639-024-12525-1

Joy, J., Balakrishnan, K., & Madhavankutty, S. (2020). Developing a bilingual mobile dictionary for Indian Sign Language and gathering users experience with SignDict. *Assistive Technology*, *32*(3), 153-160. https://doi.org/10.1080/10400435.2018.1508093

Joy, J., Balakrishnan, K., & Sreeraj, M. (2019). SiLearn: An intelligent sign vocabulary learning tool. *Journal of Enabling Technologies*, *13*(3), 173-187. [doi: 10.1108/JET-03-2019-0014]

King, M., Romski, M., & Sevcik, R. A. (2020). Growing up with AAC in the digital age: a longitudinal profile of communication across contexts from toddler to teen. *AAC: Augmentative & Alternative Communication*, *36*(2), 128-141. https://doi.org/10.1080/07434618.2020.1782988

Lawal, A., Cavus, N., Lawan, A. A., & Sani, I. (2024). Hausar Kurma: Development and Evaluation of Interactive Mobile App for the English-Hausa Sign Language Alphabet. *IEEE Access*, *12*, 46012-46023. https://doi.org/10.1109/ACCESS.2024.3381538

Lekova, A. K., Tsvetkova, P. T., & Tanev, T. K. (2021). ROBOT-ASSISTED PSYCHOSOCIAL TECHNIQUES FOR LANGUAGE LEARNING BY HEARING-IMPAIRED CHILDREN [Article]. *International Journal on Information Technologies & Security*, *13*, 63-76. https://search.ebscohost.com/login.aspx?direct=true&AuthType=shib&db=iih&AN=154408226&site=ehost-live&scope=site&custid=swinb

Majorano, M., Santangelo, M., Redondi, I., Barachetti, C., Florit, E., Guerzoni, L., Cuda, D., Ferrari, R., & Bertelli, B. (2024). The use of a computer-based program focused on the syllabic method to support early literacy in children with cochlear implants. *International journal of pediatric otorhinolaryngology*, *183*(gs2, 8003603), 112048. https://doi.org/https://dx.doi.org/10.1016/j.ijporl.2024.112048

Meinzen-Derr, J., Sheldon, R., Altaye, M., Lane, L., Mays, L., & Wiley, S. (2021). A Technology-Assisted Language Intervention for Children Who Are Deaf or Hard of Hearing: A Randomized Clinical Trial. *Pediatrics*, *147*(2), 1-10. https://doi.org/10.1542/peds.2020-025734

Meinzen-Derr, J., Sheldon, R. M., Henry, S., Grether, S. M., Smith, L. E., Mays, L., Riddle, I., Altaye, M., & Wiley, S. (2019). Enhancing language in children who are deaf/hard-of-hearing using augmentative and alternative communication technology strategies. *International journal of pediatric otorhinolaryngology*, *125*, 23-31. [doi: 10.1080/17483107.2016.1269210] [Medline: 27982714]

Meinzen-Derr, J., Wiley, S., McAuley, R., Smith, L., & Grether, S. (2017). Technology-assisted language intervention for children who are deaf or hard-of-hearing; a pilot study of augmentative and alternative communication for enhancing language development. *Disability & Rehabilitation: Assistive Technology*, *12*(8), 808-815. https://doi.org/10.1080/17483107.2016.1269210

Mohamad, N., & Hashim, N. L. (2021). UX Testing for Mobile Learning Applications of Deaf Children. *International Journal of Advanced Computer Science and Applications*, *12*(11), 294-299. https://doi.org/10.14569/IJACSA.2021.0121134

Mohammad, H., Tamimi, H., & Abuamara, F. (2022). An Educational Arabic Sign Language Mobile Application for Children with Hearing Impairment [Article]. *International Journal of Interactive Mobile Technologies*, *16*(20), 114-129. https://doi.org/10.3991/ijim.v16i20.32427

Mood, D., Sheldon, R., Tabangin, M., Wiley, S., & Meinzen-Derr, J. (2022). Technology assisted language intervention (TALI) for children who are deaf/hard of hearing: promising impact on pragmatic skills. *Deafness & Education International*, *24*(4), 334-355. https://doi.org/10.1080/14643154.2022.2135731

Navarro-Newball, A. A., Loaiza, D., Oviedo, C., Castillo, A., Portilla, A., Linares, D., & Álvarez, G. (2014). Talking to Teo: Video game supported speech therapy [Article]. *Entertainment Computing*, *5*(4), 401-412. https://doi.org/10.1016/j.entcom.2014.10.005

Parvez, K., Khan, M., Iqbal, J., Tahir, M., Alghamdi, A., Alqarni, M., Alzaidi, A. A., & Javaid, N. (2019). Measuring effectiveness of mobile application in learning basic mathematical concepts using sign language. *Sustainability*, *11*(11), 3064.

Ployjiw, U., & Michel, P. C. (2023). Development of Augmented Reality Learning Materials for the Hearing Impaired Students in Primary I. *International Journal of Information and Education Technology*, *13*(11), 1696-1703. https://doi.org/10.18178/ijiet.2023.13.11.1978

Rocha, D. F. S., Bittencourt, I. I., de Amorim Silva, R., & Ospina, P. L. E. (2023). An assistive technology based on Peirce's semiotics for the inclusive education of deaf and hearing children. *Universal access in the information society*, *22*(4), 1097-1116. https://doi.org/10.1007/s10209-022-00919-2

Shelton, B. E., & Parlin, M. A. (2016). Teaching math to deaf/hard-of-hearing (DHH) children using mobile games: Outcomes with student and teacher perspectives. *International Journal of Mobile and Blended Learning*, *8*(1), 1-17. https://doi.org/10.4018/IJMBL.2016010101

Shoaib, L., Khan, S., Abbas, M. A., & Salman, A. (2018). Enabling profound hearing impaired children to articulate words using lip-reading through software application. *Journal of the Pakistan Medical Association*, *68*(3), 432-436. https://www.scopus.com/inward/record.uri?eid=2-s2.0-85042670544&partnerID=40&md5=aee15f67d1e5eb655a6fc163837559b8

Suarsana, I. M. (2021). Developing Interactive Digital Mathematics Book with Multi Representation Approach for Deaf Students. *International Journal of Emerging Technologies in Learning*, *16*(13), 128-141. https://doi.org/10.3991/ijet.v16i13.22459

Sztahó, D., Kiss, G., & Vicsi, K. (2018). Computer based speech prosody teaching system [Article]. *Computer Speech & Language*, *50*, 126-140. https://doi.org/10.1016/j.csl.2017.12.010

Techaraungrong, P., Suksakulchai, S., Kaewprapan, W., & Murphy, E. (2017). The design and testing of multimedia for teaching arithmetic to deaf learners. *Education and Information technologies*, *22*(1), 215-237. https://doi.org/10.1007/s10639-015-9441-1

Vasel, H., & Ragonis, N. (2024). Empowering hearing-impaired students: A mobile learning intervention in Israeli Arab elementary education. *Journal of Research in Special Educational Needs*. https://doi.org/10.1111/1471-3802.12697

Véliz, S., Espinoza, V., Sauvalle, I., Arroyo, R., Pizarro, M., & Garolera, M. (2017). Towards a participative approach for adapting multimodal digital books for deaf and hard of hearing people. *International Journal of Child-Computer Interaction*, *11*, 90-98.

Yaman, F., Dönmez, O., Avci, E., & kabakçi yurdakul, i. (2016). Integrating mobile applications into hearing impaired children's literacy instuction. *Egitim ve bilim-education and science*, *41*(188). https:/doi.org/10.15390/EB.2016.6687

Zainuddin, N. M. M., Maarop, N., & Hassan, W. A. W. (2022). Measuring Satisfaction on Augmented Reality Courseware for Hearing-Impaired Students: Adjustment Formula form System Usability Scale. *Asian Journal of University Education*, *18*(2), 348-360. https://doi.org/10.24191/ajue.v18i2.17990
